# Computer-aided diagnostic for classifying chest X-ray images using deep ensemble learning

**DOI:** 10.1186/s12880-022-00904-4

**Published:** 2022-10-15

**Authors:** Lara Visuña, Dandi Yang, Javier Garcia-Blas, Jesus Carretero

**Affiliations:** 1 Department of Computer Science and Engineering, University Carlos III, Madrid, Spain; 2 Beijing Electro-Mechanical Engineering Institute, Beijing, China

**Keywords:** Deep ensemble learning, COVID-19 classification, CNN, Stacking, Voting, Grad-CAM

## Abstract

**Background:**

Nowadays doctors and radiologists are overwhelmed with a huge amount of work. This led to the effort to design different Computer-Aided Diagnosis systems (CAD system), with the aim of accomplishing a faster and more accurate diagnosis. The current development of deep learning is a big opportunity for the development of new CADs. In this paper, we propose a novel architecture for a convolutional neural network (CNN) ensemble for classifying chest X-ray (CRX) images into four classes: viral Pneumonia, Tuberculosis, COVID-19, and Healthy. Although Computed tomography (CT) is the best way to detect and diagnoses pulmonary issues, CT is more expensive than CRX. Furthermore, CRX is commonly the first step in the diagnosis, so it’s very important to be accurate in the early stages of diagnosis and treatment.

**Results:**

We applied the transfer learning technique and data augmentation to all CNNs for obtaining better performance. We have designed and evaluated two different CNN-ensembles: Stacking and Voting. This system is ready to be applied in a CAD system to automated diagnosis such a second or previous opinion before the doctors or radiology’s. Our results show a great improvement, 99% accuracy of the Stacking Ensemble and 98% of accuracy for the the Voting Ensemble.

**Conclusions:**

To minimize missclassifications, we included six different base CNN models in our architecture (VGG16, VGG19, InceptionV3, ResNet101V2, DenseNet121 and CheXnet) and it could be extended to any number as well as we expect extend the number of diseases to detected. The proposed method has been validated using a large dataset created by mixing several public datasets with different image sizes and quality. As we demonstrate in the evaluation carried out, we reach better results and generalization compared with previous works. In addition, we make a first approach to explainable deep learning with the objective of providing professionals more information that may be valuable when evaluating CRXs.

## Background

Coronavirus disease (COVID-19) is an infectious disease caused by a newly discovered coronavirus. The world is facing an unprecedented challenge with communities and economies everywhere affected by the growing COVID-19 pandemic. Globally, until 18 May 2021, there have been 163,212,543 confirmed cases of COVID-19, including 3,383,979 deaths, reported by WHO [1]. Currently, the number of cases continues to increase up to 511,252,681 including 6,238,149 deaths, until 4 May 2022 [2]. The method commonly used to detect COVID-19 is the Reverse Transcription Polymerase Chain Reaction (RT-PCR) test. However, RT-PCR test is a time-consuming, laborious, and complicated manual process [3]. A current need required now is a fast, simple-to-use, portable, and affordable early detection system for COVID-19.

Pulmonary function diagnostic is often examined by medical imaging, primarily using X-Ray, but also complemented with scan Computed tomography (CT) and ultrasound, due to the significant comparison in the lung medical image data. During the pandemic, the fatigue of the doctors was exposed, they was forced to work many hours with high pressure. Doctors and radiologists needed to diagnose many lung X-RAY per day to distinguish COVID-19 disease versus others pathologies. This situation remark the necessity of new Computer-Aided Diagnosis systems (CAD systems).

In recent years, in the field of medical image analysis, especially in radiology, deep learning techniques have been used to improved detection, diagnosis, and treatment of several diseases [4]. Since lungs X-Ray serves as the foundation for other imaging studies, using X-Rays and deep learning to diagnose COVID-19 is the predominant first option for evaluating pulmonary symptoms using imaging techniques [5–7]. Application of Convolutional Neural Network (CNN) techniques coupled with radiological imaging can be helpful in the accurate identification of this disease and can also be supportive in overcoming the issue of a shortage of trained physicians in remote communities [8]. Thus, efforts have been taken in this area, [9] present a CAD for segmentation and classification of pulmonary nodules in radiological 3D imaging .

However, any CNN model generates a certain percent of erroneous classifications, in the form of false positives and false negatives. One possibility recently suggested to minimize those errors is to use CNN ensembles [10–12] , the combination of different convolutional neural networks architectures can improve the system robustness and generalization [13, 14]. In this paper, we propose a novel architecture for a CNN-based ensemble for classifying chest X-Ray images into four classes: Viral Pneumonia, Tuberculosis, COVID-19, and Healthy. To minimize missclassifications, we included six different base CNN models in our architecture (VGG16, VGG19, InceptionV3, REsNet101V2, DenseNet121 and CheXnet) and it could be extended, which output is fed to a classifier for definitive classification. The six base CNN networks have different internal architectures to push dissimilarity in the decision process. VGG16 and VGG19 are Deep CNN, InceptionV3 as an architecture that is wider than deeper, ResNet101V2 belongs to the deep residual networks family, while DenseNet121 and CheXnet have dense blocks which are densely connected. We applied the transfer learning technique [15] to all CNNs for obtaining better performance.

The main contributions of this paper are as follows:

- Design an generalizable and functional CAD to diagnoses different diseases only with chest X-Ray (CRX). This diagnosis could be use as a first stage in the medical flow for pulmonary diagnosis.
- Creating a large dataset of COVID-19 chest X-Ray images for training, validation, and testing models. We are mixing chest X-Ray images from different subjects, so that we reach the biggest generalization.
- Designing and evaluating two different CNN-ensembles (Stacking and Voting Ensemble) for pulmonary disease classification by using CRX. To look for the best way to ensemble CNN applied to CRX.
- Proposing a model that not only achieves a high level of accuracy but also can detect misclassified chest X-Rays. Moreover, it could be helpful for doctors to detect other lung diseases (i.e., pneumonia, tuberculosis).
- Analyzing the heatmaps of the ensembles and adapting the Gradient-weighted Class Activation Mapping (Grad-CAM) algorithm to do a mixed heatmap with all the base CNNs. This system will be able to detect radiological abnormalities in CRXs, and assist doctors and radiologist in the interpretation.

This paper is organized as follows. The second section include the related work. Section “Methods” includes description of data-set, illustration of the base CNNs models, the ensemble approach proposed in this paper, the evaluation metrics, and the technique of Grad-CAM. Following sections present the results obtained with our proposed models and a discussion of it. Section “Discussion” analyzes the experimental results obtained. Finally, the conclusion remarks are presented in last section.

## Related work

This paper presented an automated computer system based on neural networks that have proved to be a fast and non-invasive way to detect several lung diseases from CRX images [16]. X-Rays are not as expensive as other medical images (i.e. PET or TAC), however specialized experts (mainly radiologists) are needed to analyze them.

By this reason, previous studies analyses the diagnosis of lung diseases, such as COVID, by using CT [17, 18]. The availability of large datasets with medical images, the advances in deep learning, and the computer power evolution have boosted the development of computer medical aid systems to assist experts to make their diagnosis [19].

Earlier studies have shownn the effectiveness of using CNNs to detect diseases. Rezaeijo, Seyed Masoud, et al. [20] detect COVID-19 using CT images, assessing the detection results using different deep learning models (DenseNet201, ResNet50, VGG16 and Xception) and combining them with machine learning algorithms (RF, SVM, DT, KNN and LGR). Obtaining the highest accuracy using CNN DenseNet201. Brunese et al. [21] proposed to use two VGG16 networks: The first one discriminates between healthy or diseased X-Rays, while the second focuses on distinguishing COVID-19 from other pulmonary diseases. They reached an accuracy of 0.96 and 0.98 respectively. This study used 6523 chest X-Ray, but only 250 were from COVID-19 patients. Alhudhaif et al. [22] proved the capacity of the CNN to differentiate COVID-19 from other types of pneumonia. The study used transfer learning with three CNNs (DenseNet, ResNet, and SqueezeNet). Its dataset included 318 COVID-19 images and 650 images from other types of pneumonia. The best performance was achieved by using DenseNet, with an accuracy of 94.96%.

The capacity of multiple CNNs for detecting tuberculosis was presented by Rahman et al. [23]. This study also analyzed the advantages of using pulmonary segmentation, reaching their best results using DenseNet201 with a previous segmentation of the CRX. Rangarajan et al. proposed in [24] an AI-based system for COVID diagnosis. The study analyzed five pre-trained CNNs and then deployed the two better models in a smartphone. The better performance was for VGG16, with an accuracy of 98.6%. This study used a not well-balanced dataset, with fewer COVID-19 images. To balance the data, the authors applied GAN networks and data augmentation.

Several research groups have also recently published deep learning ensemble strategies for pulmonary disease detection by using CRX [25–28]. In the results section, Table 6 shows a comparative study of our enhanced CNN ensemble with the aforementioned works.

## Methods

### Proposed architecture

**Fig. 1:**
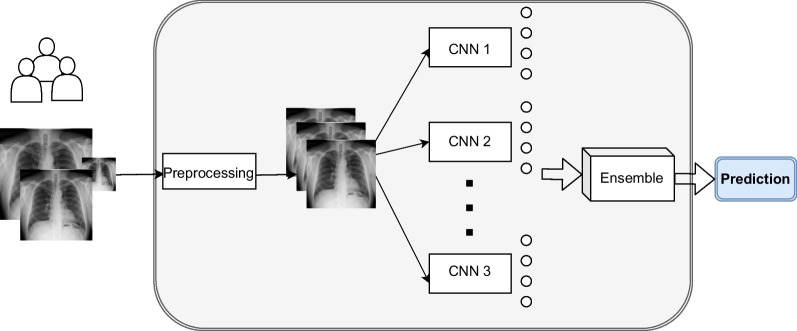
Proposed architecture of computer-aided diagnosis system based on CNN-ensemble

Figure 1 shows the architecture of the ensemble implemented in this work. This architecture has three stages: preprocessing, CNN classification, and ensemble classification.

Preprocessing stage enables to homogenize images coming from different datasets by resizing and rescaling the input images. After preprocessing, images are fed to the base CNN models that run in parallel to classify images. As explained before, we have included six base CNNs, with different models and architectures. Thus, we ensure dissimilarities in classification. After this stage, we have six classification results, which are fed to the ensemble. In the last stage, the ensemble receives all the previous inputs and finally produces the definitive classification of the image. In this work, we have implemented and evaluated different ensembles, Voting and Stacking, as explained in this section.

### Dataset description

One of the main objectives of this work is to use a large number of images for both training and testing models. With a large dataset, we can reach a bigger generalization as the dataset has a wide range of CRX from different subjects.

**Fig. 2:**
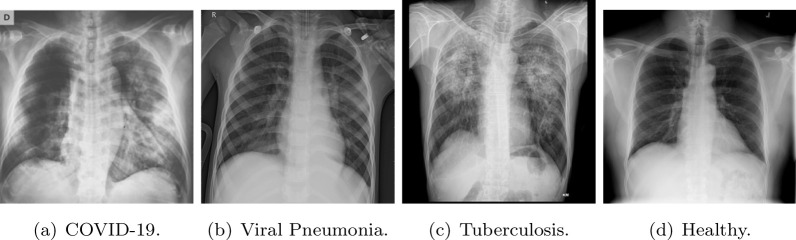
CRX examples from the dataset

Our dataset has 11,954 chest X-Ray radiographies labeled with different pulmonary diseases or labeled as healthy (images without lung pathology). The dataset is not balanced, this imbalance is due to the lower number of viral pneumonia radiographies. The database is composed of 3616 COVID-19, 1345 viral pneumonia, 3493 tuberculosis, and 3500 healthy images. In order to facilitate the reproducibility of this work, our CRX database is composed of available databases. Additionally, we have included images with considerable quality and other that comes even from mobile phones photographs to harden the detection capacity of the models. Figure 2 shows examples of images of the dataset for the different diseases including a healthy subject.

Both healthy and tuberculosis images are from “Tuberculosis (TB) Chest X-Ray Database” [29] collected by researchers from Qatar and Dhaka University, Doha, Qatar, and collaboration with doctors from Hamad Medical Corporation and Bangladesh. All the images are CRX in PNG format and a size 512 $\times$ 512. The COVID and Pneumonia images are also from a Kaggle dataset: “COVID-19 Radiography Database” [30], which collects images from different sources. This dataset was collected by the same researchers as the “Tuberculosis (TB) Chest X-Ray Database”. All the images in this database are CRX in PNG format and a size 256 $\times$ 256. An assembled version of the dataset is provided.^1^

For the training and evaluation of the system, we use a hold-out cross-validation scheme. We divided the complete dataset in three datasets. Table 1 shows how we divided the dataset into train, validation, and test subsets. First, we allocated 25% of the images of each class for testing, to prove the results of our model. The remaining 75% was randomly split again between train (80%) and validation (20%). The training images were the only ones that we used to train our models, while the validation split was used for tuning the hyperparameters and selecting the best base-CNNs for the ensembles.

**Table 1:** Dataset split into train, validation, and test

	Total CRX	Train	Validation	Test
COVID-19	3616	2170	542	904
Healthy	3500	2100	525	875
Pneumonia	1345	808	201	336
TB	3493	2097	524	874
Total	11,954	7175 (60%)	1792 (15%)	2989 (25%)

### Pre-processing of the images

Due to the heterogeneity of the images’ sources, there were images of different sizes. Thus, first, we resized all the X-Ray images to $256\times 256$ to homogenize the input for the different CNN models. Additionally, all images were normalized into a 0–1 range to narrow down the values of the images and accelerate the neural network. The radiography images are in grey scale (1-channel images), while the base-CNNs expect for 3-Channel images. By this reason, we compose false-RGB images, replicating the grey-scale information in every image channel.

For the training of the base CNNs, we applied data augmentation using the *ImageDataGenerator* of Keras tool, which is designed for real-time data augmentation. With this tool, images are randomly rotated, shifted, and zoomed in every epoch and for every CNN. Thereby, every epoch all the images used to train the Base-CNN are changed. Data augmentation process avoids overfitting, enlarges the dataset, and improves the generalization of our models.

### Proposed convolutional neural network for base classifiers

For this work, we have chosen six different base CNN architectures trying to obtain different features of every image: VGG16, VGG19 [31], InceptionV3 [32], ResNet101V2 [33], DenseNet121 [34] and CheXnet [35]. Each CNN worked completely independently of the other. Each base model was trained with the original data, learned and fit from the images, to issue a prediction. After running all them in parallel, their predictions were introduced into the ensemble models to obtain the final prediction.

The first two CNNs (VGG16 and VGG19) are deep networks that employ small convolutional filters ($3\times 3$) followed by maxpool layers. These CNNs have 16 and 19 layers respectively. These CNNs have shown a great performance in other related works [21]. InceptionV3 belongs to a family of networks that use a 48 layers architecture that is wider than deeper. InceptionV3 uses specific blocks that take the output of one layer, uses different convolutional filters at the same level, and concatenates the results into the next layer. ResNet101V2 belongs to the deep residual networks family. It employs residual blocks, which take one previous signal, skip some layers, and then this signal is added later in the network. ResNet101V2 has 101 layers and is the network with more weights used in this work, which results in a longer time for training. The last two base CNNs classifiers are DenseNet121 and CheXnet, which have the same architecture with 121 layers. These networks have dense blocks which are densely connected, all layers are connected inside each block. The difference between the two CNNs classifiers is their weights. CheXnet was trained with 112,120 frontal-view chest X-Rays with 14 different thoracic anomalies.

All the base CNNs classifiers are used with previous learning. This technique is called “Transfer Learning”, which takes advantage a neural network with previously training. In our case, we considered the weights of each network obtained with the ImageNet dataset [36]. This dataset is a classification challenge with more than 12 million images and 1000 different classes. CheXnet manages the weights reached in the training with the 112,120 CRXs. By including CheXnet, we want to study the impact in classification and generalization when we use a pre-trained network with a similar dataset to the current one.

### Convolutional neural network classifiers implementation details

All the base-CNNs were trained with the same training images during 100 epochs using a batch size of 16. In each epoch, every image was randomly modified with the *ImageDataGenerator* defined previously. We also defined an early stopping with patience of 20 epochs, which monitors the accuracy of the training data. During the training, we froze the convolutional weights so that only the dense layers were trained. We selected the categorical cross-entropy (see Eq. 1) as loss function and the Adam Optimizer from Keras with the learning rate of 0.001. All those hyperparameters were selected taking into account the validation results of the base model.

1 $$\begin{aligned} \textit{Categorical Cross-entropy}= -\sum _{i}^{NClases} Target_i \cdot \log (Predictions_i) \end{aligned}$$

As our dataset is not well-balanced, we decided to weight the loss function to assist with the network training. Equation 2 assigns a different weight to every class (c), so that a big weight is assigned to the class with fewer images and a small weight is assigned to the class with more images. This makes the model pay more attention to the classes under-represented.

2 $$\begin{aligned} Weight Class (c) = \frac{1}{2} \cdot \left( 1- \frac{{N^{\underline{O}}\,\, images \,\, belong \,\, to \,\, the \,\, class \,\, c} }{{N^{\underline{O}} \,\, total \,\, of \,\, images}} \right) \end{aligned}$$

### Architectural details of the proposed Metamodels

The proposed architecture provides two ensemble alternatives : a simple voting (Voting Ensemble) and a deep learning based ( Stacking Ensemble) ensembles. Ensembles are metamodels, which combine the predictions of the base models to obtain the final prediction.

The approach for the Voting Ensemble consists of selecting the most frequent classes by the CNN base, following a majority vote. First, the classifications are predicted by all six base models, executed, and collected in parallel. Those classifications are fed as inputs to the ensemble. A vote indicates that an image belongs to a specific class. The most frequent voted class is considered by the ensemble as the predicted one. In the case that two or more classes have the maximum vote frequency, the ensemble selects the class that has been voted for the CNN model with the highest validation accuracy. The algorithm for the Voting Ensemble is shown in Algorithm 1.
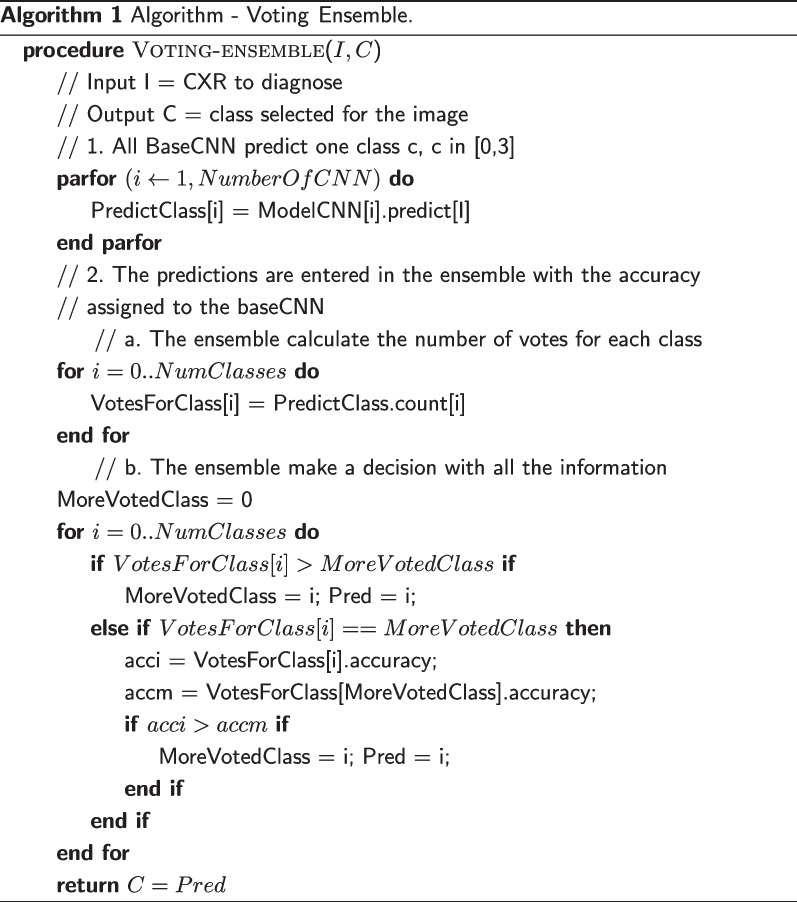


With this Voting Ensemble, we avoid the errors due to X-Ray images that could be misclassified by one base CNN, but not by all them. This ensemble emulates the case where the situation is not clear for a doctor or a specialist and she needs to be questioned by a whole team to conclude the diagnosis.

**Fig. 3:**
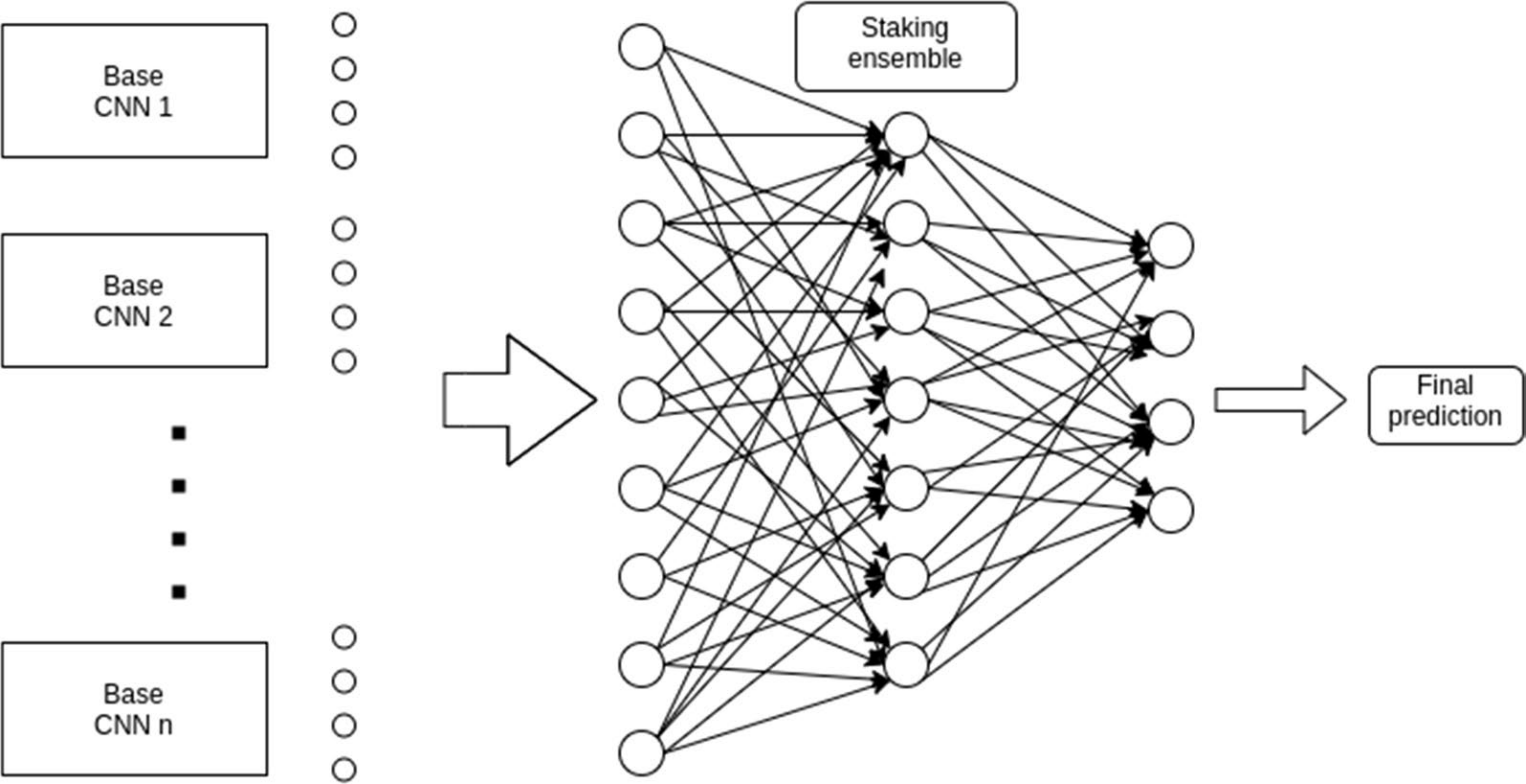
Stacking Ensemble architecture

For the second approach, Stacking Ensemble, we designed a dense neural network to find the best way for combining the base CNN predictions. The learning ensemble is designed with 3 layers with 24, 12, and 4 neurons, and a dropout of 0.1 between them. The first two layers use *relu* as an activation function and the last layer uses a *softmax*. The output of the base classifiers models for an image are stacked and used as input for the learning ensemble (see Fig. 3). This ensemble learns from the base model outputs to make its final prediction.

In this case, we take advantage of the different percentages that the base CNNs assign, not only to the selected class, but also to the rejected ones. This step is done to check if our Stacking Ensemble approach can extract new and better conclusions from this information.

### Metamodels implementation details

For the Stacking Ensemble, we stacked all the predictions of the base models and trained the ensemble during 100 epochs with a batch size of 16. The optimizer selected was Adam (learning rate of 0.001) and the mean squared error(see equation 3) was selected as a loss function. We only need to train the Stacking Ensemble, for the Voting Ensemble, we only needed to correctly join all predictions of the base models without previous training.

3 $$\begin{aligned} \textit{MSE}=\frac{1}{NClases} \cdot \sum _{i=1}^{NClases}( Target_i - Predictions_i)^2 \end{aligned}$$

### Performance evaluation metrics

To evaluate the performance of the different CNN and ensembles, we use metrics such as accuracy, precision, recall, and F1. The accuracy is an intuitive way to assess the global performance of the system by dividing the total of the well-classified samples between the total number of samples:

4 $$\begin{aligned} Accuracy(ACC) = \frac{CorrectPredictions}{ TotalSamples} \end{aligned}$$

Our study is based on a multiclass classification so, it’s very important to make the evaluation of each class. For this assess we employ the following metrics:

5 $$\begin{aligned} Precision(P)= & {} \frac{TP}{TP + FP} \end{aligned}$$

6 $$\begin{aligned} Recall(R)= & {} \frac{TP}{TP + FN} \end{aligned}$$

7 $$\begin{aligned} F-measure(F1)= & {} \frac{2 \cdot Precision \cdot Recall}{Precision + Recall} \end{aligned}$$

where true positives (TP) are the images well predicted into their class, true negatives (TN) are the images correctly denoted as not belonging to the class, false positives (FP) are the samples predicted wrongly as a different class, and, finally, false negatives (FN) are the samples that, although they belong to the class being studied, are classified in another. All these metrics can be extracted elaborating the confusion matrix, which represents how the system predicted the samples into the different classes, representing the actual value versus the predicted value. We also construct confidence intervals (CIs) with the boostrap method.

For evaluation of the diagnosis significance, we provide the Receiver Operating Characteristic (ROC) and the Precision-Recall curve (PR curve). We also use T-distributed Stochastic Neighbor Embedding (t-SNE) transformation for visualizing the data in two-dimensions.

### Attention heatmaps

Another technique to evaluate convolutional neural networks is Grad-CAM [37]. This technique is a visual method to check that the CNN is not biased by the training images and is looking at the lung area to carry out predictions. The Gradient-weighted Class Activation Map was presented in [37]. It uses the gradient flowing into the last convolutional layer to localize and highlighting the areas more important for the classification of an image. This technique can be used with any type of CNN, which is very important for our study, because we can compare the performance of the different types of CNN used in the ensemble.

Grad-CAM provides us a visual explanation of our system decision, letting us know the parts where models are paying more attention to the classification. As we use six different convolutional networks, we need to build individual heatmaps. To analyze the complete ensemble, we aggregate all the heatmaps to conform a single image. By using this method, we can reduce the overfitting of the training data, as we can visually test if our system is biased for any external factors. Furthermore, this understanding of the network could be useful for experts to make decisions about the diagnostic.

## Results

### Convolutional neural network classifiers performance analysis

The result of the Base-CNN based on validation images is shown Table 2. In this table, we can compare the recall, precision, and F1-Score for every CNN and class. Furthermore, the table presents the global accuracy for every CNN. We achieved an accuracy greater than 90% in all the base-CNNs taking into account the validation images.

**Table 2:** Base-CNN individual performance based on validation images

		Precision	Recall	F1-score	Accuracy
VGG19	COVID-19	0.95	0.92	0.94	0.96
	Healthy	0.95	0.98	0.97	
	Viral pneumonia	0.95	0.99	0.97	
	Tuberculosis	0.96	0.95	0.96	
VGG16	COVID-19	0.98	0.89	0.93	0.95
	Healthy	0.9	1.00	0.94	
	Viral pneumonia	0.95	1.00	0.97	
	Tuberculosis	0.99	0.95	0.97	
ResNet101V2	COVID-19	0.98	0.91	0.94	0.95
	Healthy	0.89	0.99	0.94	
	Viral pneumonia	0.97	1.00	0.98	
	Tuberculosis	0.98	0.93	0.95	
DenseNet121	COVID-19	0.99	0.89	0.93	0.95
	Healthy	0.9	0.99	0.94	
	Viral pneumonia	0.91	1.00	0.95	
	Tuberculosis	0.98	0.94	0.96	
CheXnet	COVID-19	0.96	0.91	0.93	0.93
	Healthy	0.88	0.98	0.93	
	Viral pneumonia	0.97	1.00	0.98	
	Tuberculosis	0.95	0.88	0.91	
InceptionV3	COVID-19	0.96	0.89	0.92	0.92
	Healthy	0.85	0.99	0.92	
	Viral pneumonia	0.96	0.99	0.97	
	Tuberculosis	0.96	0.86	0.91	

The highest accuracy reached belongs to the VGG models, 0.96 for VGG19 and 0.95 for VGG16. There is a slight difference between the performance of the ResNet101V2, DenseNet121 and VGG16. All them achieved an accuracy of 0.95. That slight difference is due to the dissimilar classification of the different classes (COVID, Healthy, TB or Pneumonia). The worst accuracy was for CheXnet and InceptionV3 with accuracy 0.93 and 0.92 respectively. The CheXnet and DenseNet121 have the same architecture. However, DenseNet121 was trained with general images and CheXnet with X-Ray images, so they have different weights. We highlight that the specific training images for the convolutional part lack of have a positive impact on the final classification. This is motivated by the general features, which are more useful for the classification of diseases.

We evaluated newer CNNs to be sure of making the best base-CNN selection. The first one is EfficientNet [38] the second CNN evaluated was MNasNet [39], The CNNs were training the same way as our base-CNN. The EfficientNetB7 was not adequate for our classification problem, despite their good results with ImageNet and CIFAR-100 [38], this CNN classifies all the validation images as Tuberculosis, for this reason was discard for the ensemble. For the MNasNet the result were similar to base-CNN (Table 3).

**Table 3:** MNasNet performance based on validation images

		Precision	Recall	F1-score
MNasNET	COVID-19	0.97	0.93	0.95
	Healthy	0.99	0.99	0.99
	Viral Pneumonia	0.85	1.00	0.92
	Tuberculosis	0.98	0.95	0.96

MNAsNet reflects low precision than the selected Base CNN for Viral Pneumonia. Viral Pneumonia is the smallest class and these bad results can affect the whole ensemble’s performance. This classifier was discarded for this reason.

**Fig. 4:**
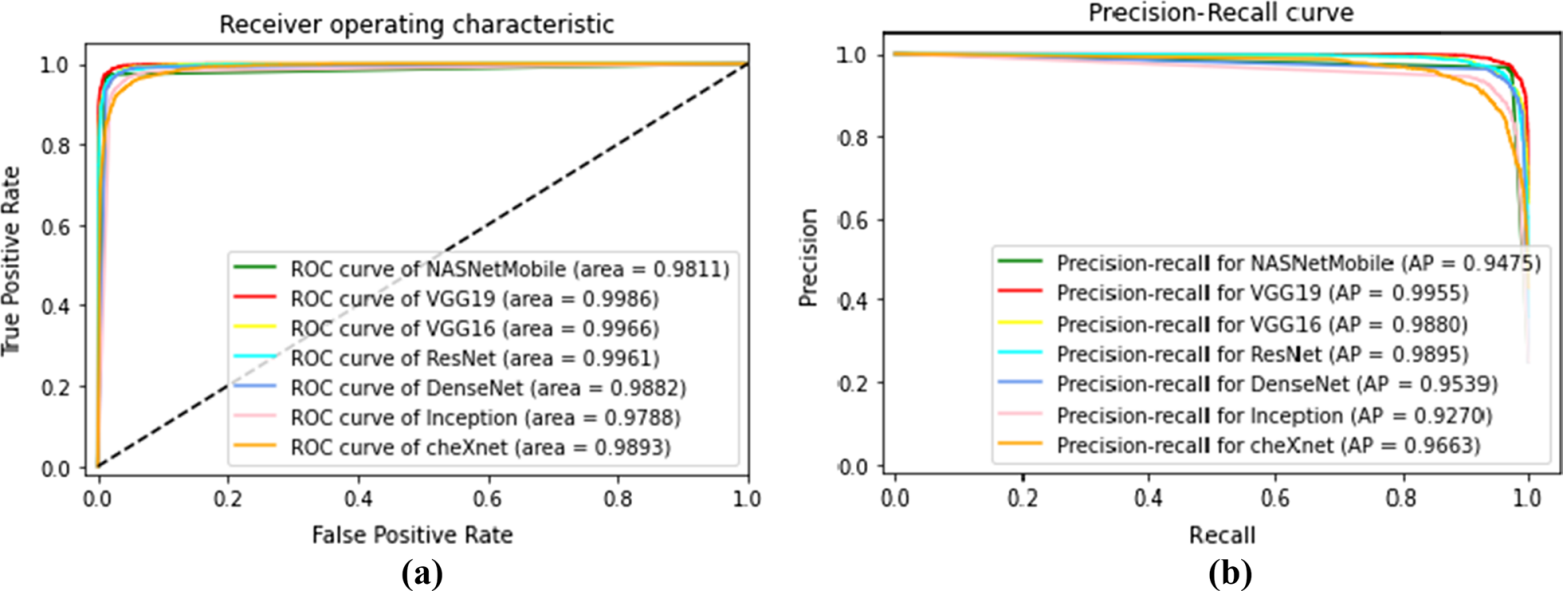
Receiver operating characteristic (ROC) and precision-recall curve (PR) for the CNN (micro-averaging)

Figure 4 contrasts the ROC and PR curves for the base CNN and MNasNet. ROC, as well as the PR curve, are metrics for binary classification, so we evaluate every CNN with compute micro-averaging ROC and PR. The micro-averaging reflects a global assess of the system.

### Aggregate heatmaps interpretation

All the models have very good performances with the pneumonia classification, with a recall near to 1 in all the base models.

We analyze the possibility of overfitting to discard the existence of artifacts in the lungs that the network could be learning. To evaluate this, we extract Grad-CAM heatmaps.

**Fig. 5:**
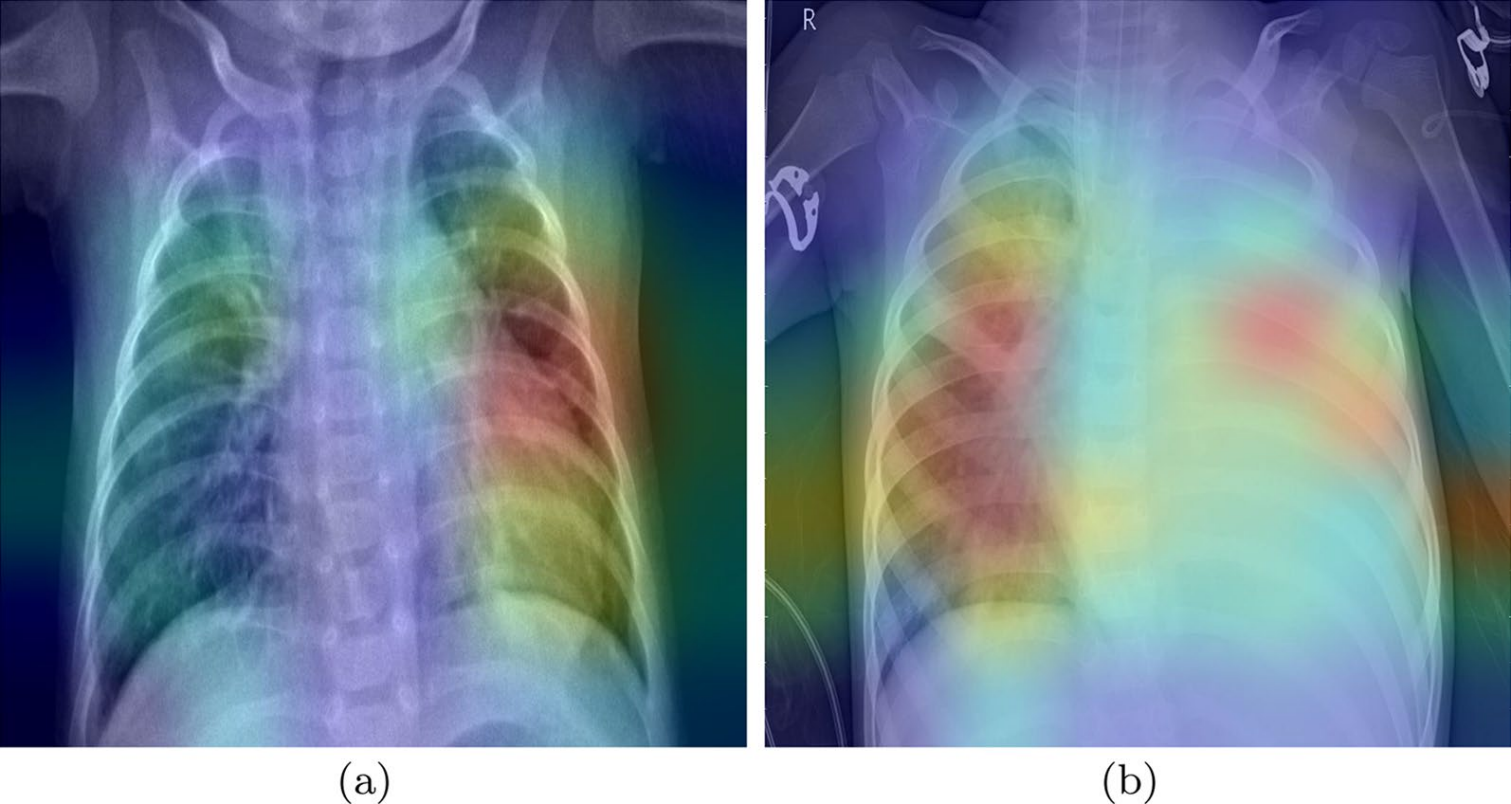
Grad-CAM heatmaps for pneumonia CRX images

The heatmaps highlight the parts of the image where the models pay more attention. The red color highlights the more important parts for the decision, the yellow and light blue colors point out areas with less importance. The images in Fig. 5 are the heatmaps of two CRX images with a diagnosis of pneumonia. We can see that the models are focusing inside the lung area to make decisions. In the left photo (a), we can observe that not all attention is inside the lungs. We have checked that only a few images look outside the chest. Thus, we can conclude that the various marks outside the lungs area are not decisive factors to make the decision.

To analyze more deeply the CNNs behavior, we compared the places of the X-Ray that our system finds important for the classification with the actual radiological findings. With this aim, we pass new images marked by experts through the CNNs. The images for this analysis are from [40], a study that discusses the radiologic appearances of tuberculosis. The images selected show the localization of some radiological marks, in addition, we can know the causes of these. These images include arrows that could influence the final heatmaps.

**Fig. 6:**
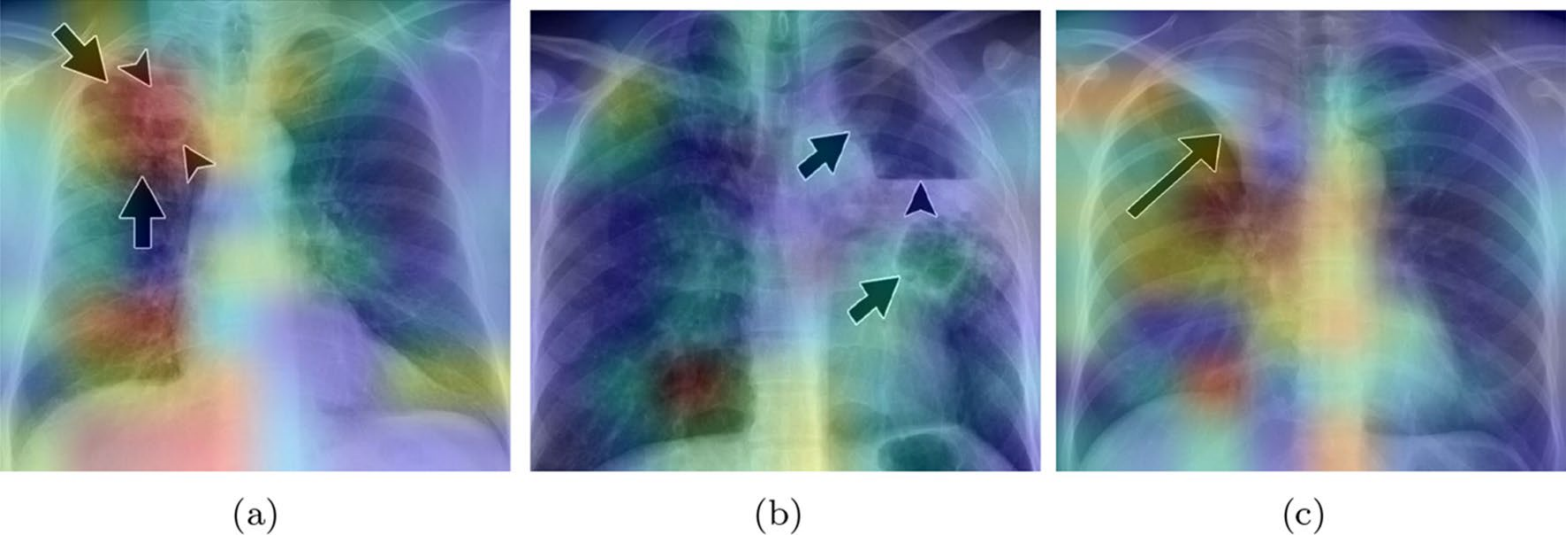
Grad-CAM heatmaps for tuberculosis CRX images with radiological findings

The first image (Fig. 6a) belongs to a 50-year-old man with tuberculosis. The little arrows point to a cavitary lesion, and the big arrows point out airspace opacities, these anomalies are well localized by our base-CNNs as shown by areas in red color.

In the second image (Fig. 6b), the heatmap provides less valuable information. The big arrows point out new cavitary lesions, our system only signs the down lesion, which is the biggest one. According to [40], the little arrow points out a large lesion with an air-fluid level, this last lesion is not found by our system.

The last image (Fig. 6c) belongs to a 41-years old woman. The arrow points out a lobe collapse. In this case, our system is focusing at the radiological finding, but it is also looking at other places in the image. This could be due to the other affections of the patient under study. The 41 years old woman has an airway involvement with tuberculosis. [40] also say that the central bronchi show an irregular thickening. These anomalies are localized by our network, getting more importance for our system than the one singed by the arrow.

Even though not all anomalies were found, we can conclude that the system learned for its own to focus on lung anomalies. The unnoticed anomalies could be explained by the fact that arrows were in the image, the insufficient quality of the images, or like in the last case, the accumulation of anomalies in a single X-Ray. This analysis is the first approach. To enhance the system to detect all the anomalies, we need to do more specific training with the support of qualified experts (radiologists and doctors) to validate the results.

### Validation of the proposed ensemble system

In this section we analyze the performance of the ensembles based on validation images. Table 4 shows the results reached with the validation data. The numbers prove the better performances of the ensembles over the base classification models. We can also observe that using the deep learning ensemble has a good repercussion in the results. This model reached an accuracy of 0.98 versus the 0.97 accuracy of the Voting Ensemble. Anyway, those results are similar or better than other state of the art solutions [10, 41].

**Table 4:** Ensembles performance based on validation images

		Precision	Recall	Score-F1	Accuracy
Stacking Ensemble	COVID-19	0.99	0.96	0.98	0.98
	Healthy	0.98	0.99	0.99	
	Viral Pneumonia	0.99	1.00	0.99	
	Tuberculosis	0.98	0.99	0.98	
Voting Ensemble	COVID-19	0.98	0.94	0.96	0.97
	Healthy	0.95	1.00	0.97	
	Viral Pneumonia	0.97	1.00	0.98	
	Tuberculosis	0.99	0.96	0.98	

**Fig. 7:**
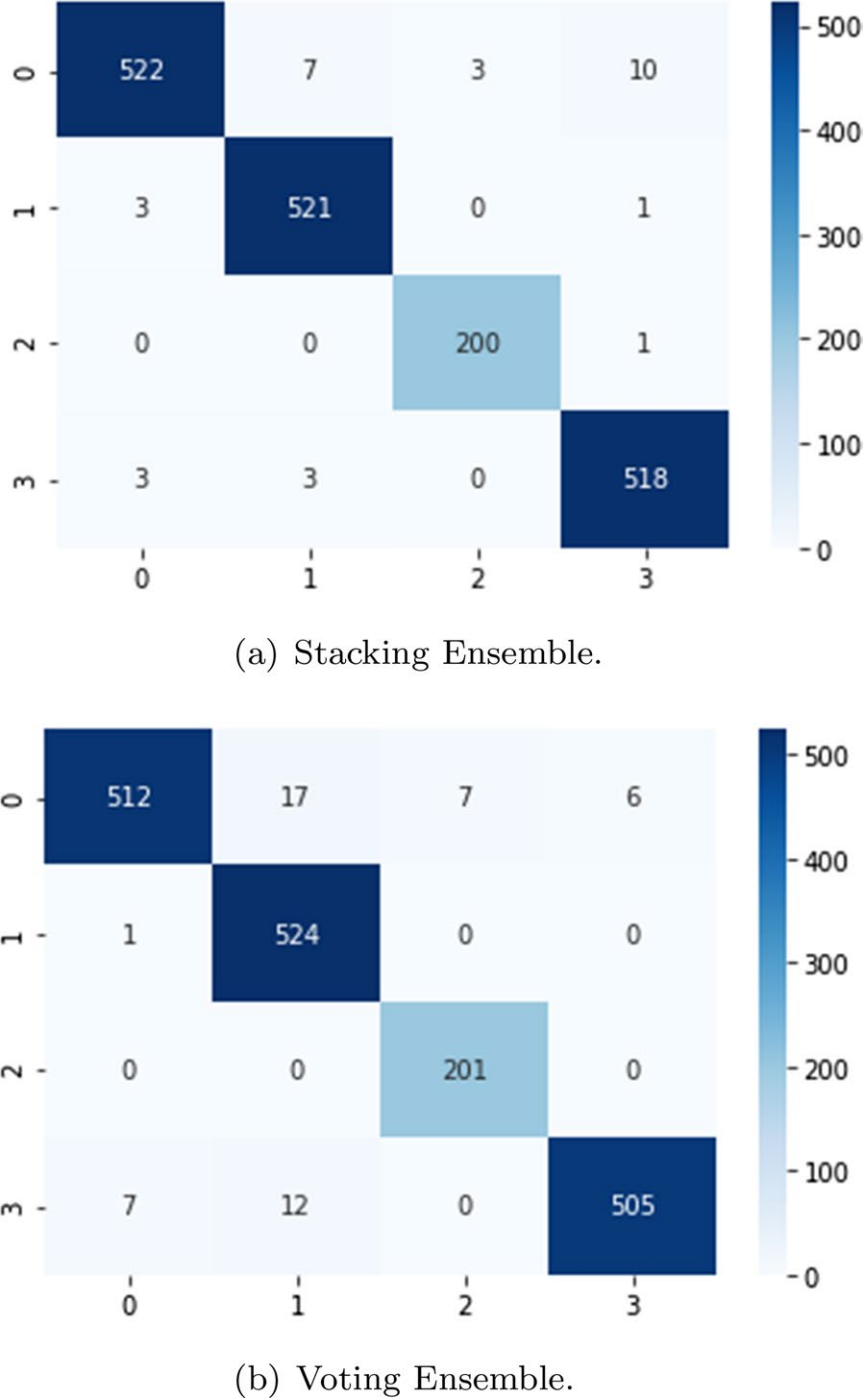
Validation confusion matrix (classes: 0-COVID-19, 1-Healthy, 2-Pneumonia, and 3-Tuberculosis)

In Fig. 7 the confusion matrix of the ensembles are shown. The Stacking Ensemble (**a**) misclassified 31 images from the 1,792 validation images, less than 2% of the images. The most common error for this ensemble is to confuse CRX labeled as COVID with tuberculosis or healthy CRX. In the case of the Voting Ensemble (**b**), 50 images are misclassified, only 2.8% of the validation images. In this ensemble, the major error is also the misclassification of the COVID images.

**Fig. 8:**
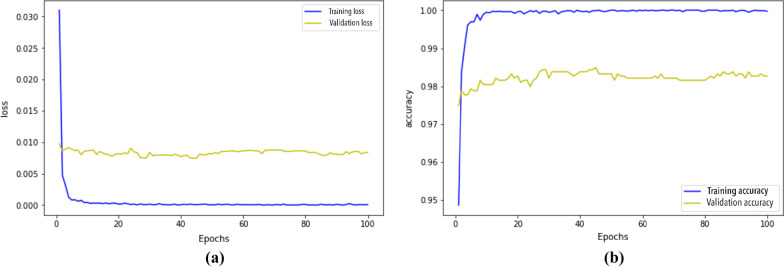
Training/validation loss and accuracy graph for the stacking ensemble

Figure 8 plots the evolution of loss and accuracy for the Stacking

Ensemble during the 100 epochs. We notice a slight overfitting as the validation loss is 0.008 upper than the training loss. The ensemble accuracy for the validation data is only 0.02 lower than training data. The performance is similar for the validation and train data, this is an advantage of using a set of different CNN.

### Performance analysis of the proposed system

To depthly analyze the behavior of the ensemble, we employed the 2989 test image set, which were never used to train or tune hyperparameters. Table 5 depicts that the performance is even better than for the validation images, reaching an accuracy of 0.99 for the Stacking Ensemble and 0.98 for the Voting Ensemble. Also, we use the bootstrap method, with 1,000 bootstrap samples of the test set to construct the CIs around the accuracy of the ensembles. The Voting Ensemble has a median accuracy of 0.98 with a 95% CI of [0.97,0.98] and the Stacking Ensemble median accuracy is 0.99 with a 95% CI of [0.98,0.99].

**Fig. 9:**
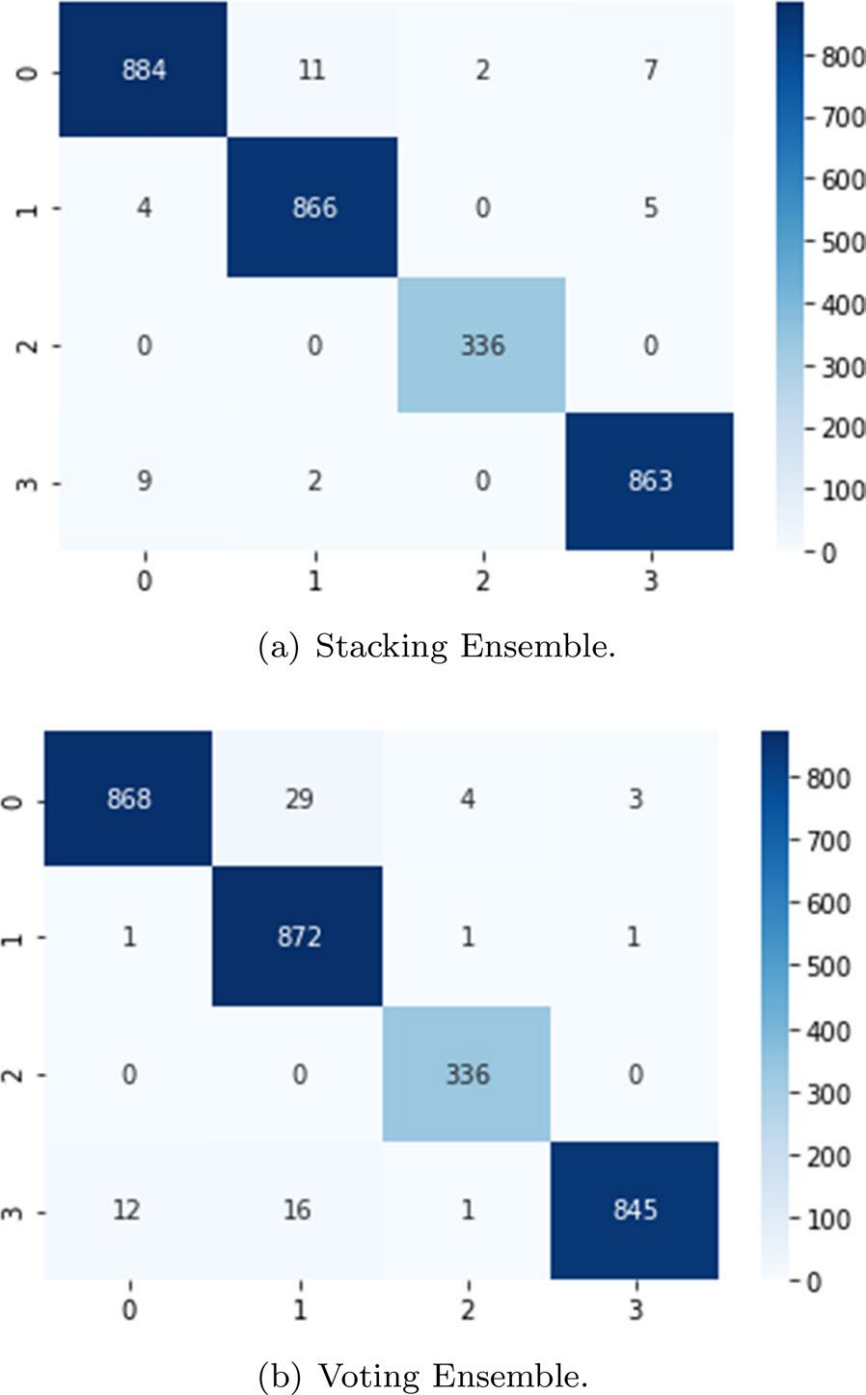
Test confusion matrix (classes: 0-COVID-19, 1-Healthy, 2-Pneumonia, and 3-Tuberculosis)

Figure 9 plots the confusion matrix of the ensembles. As may be seen, the behavior of the ensembles is similar using the validation and test images. In this case, the Stacking Ensemble misclassified 40 images (1.34% of the test images) and the Voting Ensemble 68 (2.38% of the test images).

**Table 5:** Ensembles performance based on test images

		Precision	Recall	Score-F1	Accuracy
Stacking Ensemble	COVID-19	0.99	0.98	0.98	0.99
	Healthy	0.99	0.99	0.99	
	Viral Pneumonia	0.99	1.00	1.00	
	Tuberculosis	0.99	0.99	0.98	
Voting Ensemble	COVID-19	0.99	0.96	0.97	0.98
	Healthy	0.95	1.00	0.97	
	Viral Pneumonia	0.98	1.00	0.99	
	Tuberculosis	1.00	0.97	0.98	

We notice that the ensembles mostly confuse the same images collection. We analyzed a set of 32 images that are misclassified in both ensembles , with the objective of increasing our knowledge about the metamodels. The most misclassified images are the images labeled as COVID-19. Analyzing those images, we found two main causes. First, some images lack of quality, causing bad interpretation from the system. This problem appears in almost all classes. We could solve it by doing a deeper data sanitizing but we would lose flexibility in the models to classify new data. The second problem is that some images labeled as COVID-19 show a very clear lung area, almost without signs of disease. These images are mostly classified as healthy by our system. To analyze this in a better way, we would need to have access to metadata or clinical history. It could be possible that these images belong to asymptomatic patients, who normally show very low signs of the disease. If it was the case, we could solve the problem adding more chest X-Ray belonging to asymptomatic subjects to our dataset.

**Fig. 10:**
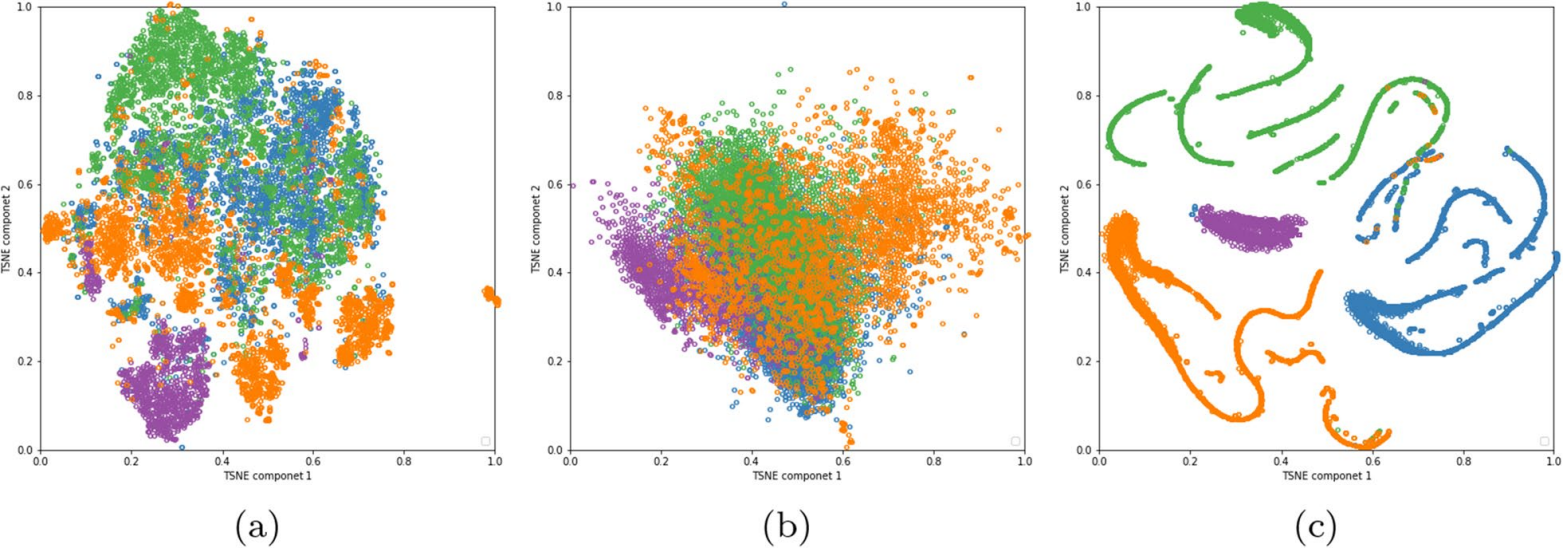
TSNE visualization for train and validation set. **a** Originals radiography images, **b** intermediate layer VGG19 (block3—pool), and **c** last layer VGG19 (dense layer). Classes: Blue-COVID-19, Green-Healthy, Purple-Pneumonia, and Orange-Tuberculosis

Figure 10a plots the t-SNE projection into two dimensions for the original data . We can affirm that the green (Healthy) and Blue (COVID) cases are the images more nearby, although, all classes are mixed.

Figure 10b, c shows the t-SNE projection into two data dimensions in the base classifier with higher accuracy (VGG19). In the intermediate layer (b), the classes are mixed in a higher way, mainly due to use a transfer learning scheme. In ( c), we observe how CNN assists the discrimination of the four classes. Despite the high accuracy of this base CNN, the clustering of the classes is not clear in some areas, justifying the use of the ensemble to combine the power of different CNN to reach better results.

**Fig. 11:**
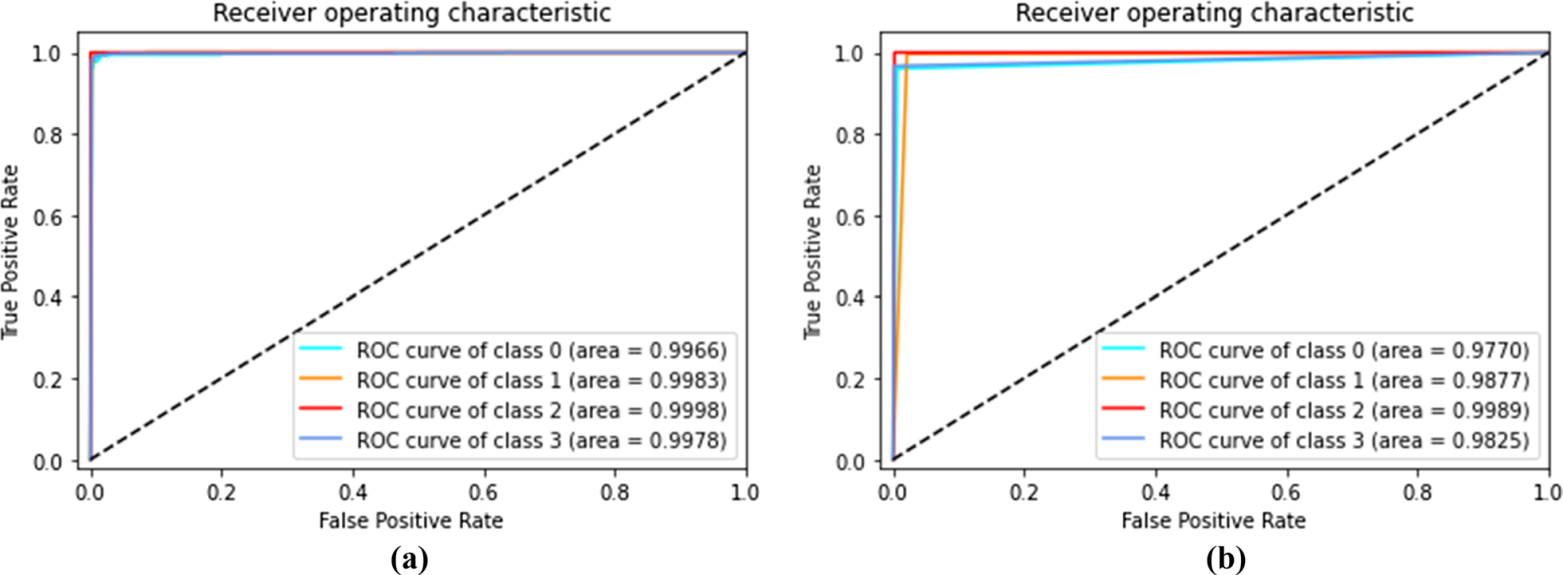
Receiver operating characteristic (ROC) for stacking ensemble (**a**) and voting ensemble (**b**) (classes: 0-COVID-19, 1-Healthy, 2-Pneumonia, and 3-Tuberculosis)

**Fig. 12:**
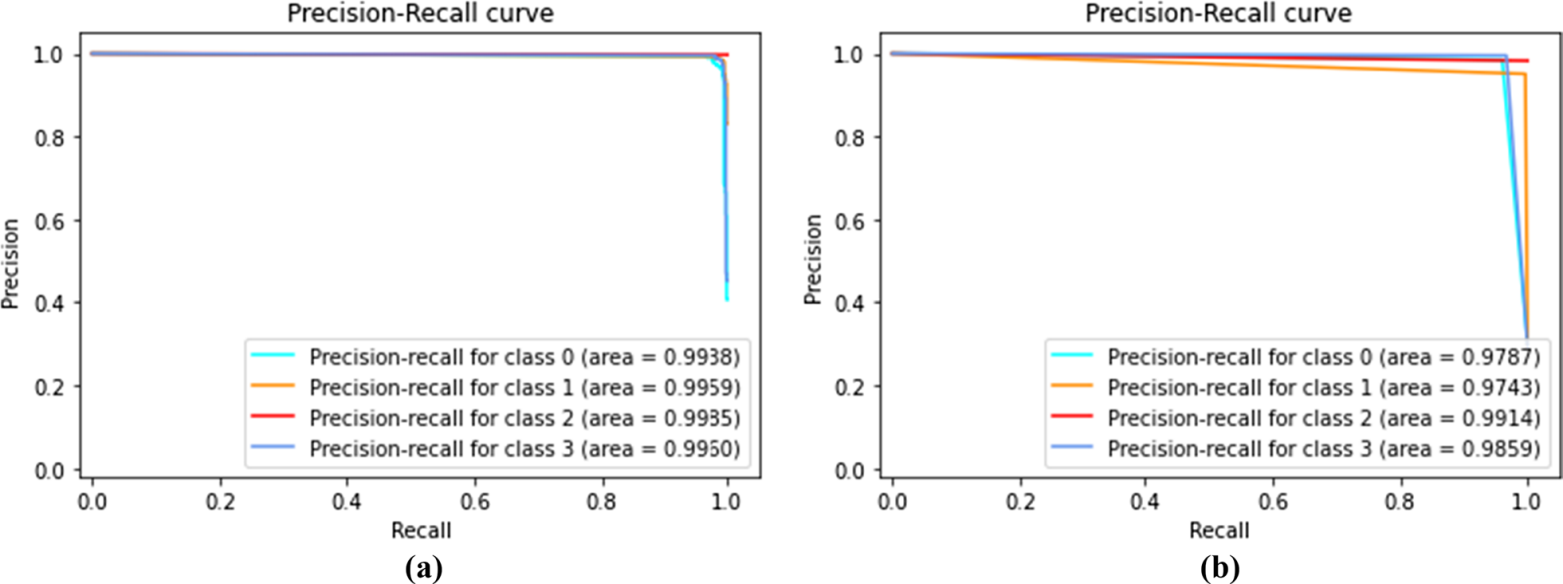
Precision-recall curve for stacking ensemble (**a**) and voting ensemble (**b**) (classes: 0-COVID-19, 1-Healthy, 2-Pneumonia, and 3-Tuberculosis)

For evaluation of the ensembles , we provide the Receiver Operating Characteristic (ROC) (Fig. 11) and the Precision-Recall curve (PR curve) metrics (Fig. 12). The ROC as well as the PR curve are metrics for binary classification , therefore, we analyse every class individually. Additionally, it is important to note that the Voting Ensemble does not use probabilistic methods, so its curves are approximation. The curves illustrate the good performance achieved by the ensembles on the test set, with an AUC very near to 1 for every class.

### Performance comparison with previous CRX ensembles

**Table 6:** Comparative study of previous ensembles for pulmonary diseases detection using Chest X-Ray images

References	Model	Clases	ACC (%)
[42]	Ensemble-CNNs (ResneXt50, DenseNet161, and InceptionV3)	3, 5	81, 88
[43]	Ensemble1-CNNs( VGG16 and DenseNet201), Ensemble2-CNNs (VGG16 and ResNet152V2)	3, 2	99, 96
[44]	Ensemble-CNNs (Resnet50V2, DenseNet201, and InceptionV3)	2	91
[7]	Decision-Tree-ensemble (ResNet18)	4	92
[45]	Ensemble-CNNs (InceptionResnet-V2, DenseNet121, and InceptionV3)	2	94
Our stacking ensemble	Ensemble-CNNs (VGG19, VGG16, ResNet101V2, DenseNet121, CheXnet, and InceptionV3)	4	99
Our voting ensemble			98

Table 6 depicts the ability of the CNN ensembles in the recognition of pulmonary diseases by using X-Ray. Our proposed ensemble obtains a better accuracy performance and a larger dataset than those in the previous studies. For example, in [43] , authors determined an accuracy of 0.99, very near to ours. The study defines an ensemble for classification between pneumonia, COVID-19, and healthy . However, we can stand out that they only use 1604 CRX, much fewer images than the 11,954 CRX used in our case.

Our system shows a better accuracy that the previous studies also, the ensembles was tested in a larger dataset, that the previous studies with similar accuracy. Also the system is design not only to discriminate between two classes, is capable to detect COVID, pneumonia, tuberculosis, and healthy CRX. We expect to enlarge the number of diseases to develop a complete computer-aid diagnosis system to detect pulmonary diseases with CRX.

We ensemble six different CNN inducing a higher computation time. Results are compared in the Table 7 including the computation time of the system. The execution time is only reported in [42], which specifies 0.077s (3 classes) and 0.135s (5 classes). These yields are comparable to our system, even though their ensemble only assembles three CNN.

**Table 7:** Execution time of the ensembles and the aggregate heatmaps generation (in seconds)

Height	Voting ensemble	Stacking ensemble	Aggregate heat-map
Single image	0.156	0.209	3.289
Validation dataset	310.156	310.267	–

Finally, Table 7 shows the time needed to generate the heatmaps from the original image. As mentioned, heatmaps can be extracted on demand to see the areas in which the system pays more attention to make the classification decision. This can be useful , especially, in case of doubtful or ambiguous images. We conclude that the assisted diagnosis system is of great value for the simultaneous analysis of large sets of images, such as in screening cases or pandemic situations such as that derived from COVID-19. The system enables a first diagnosis to be issued without the need to wait for biological tests ( i.e., PCR for COVID-19).

## Discussion

This study has presented a system of two different CNN-ensembles for pulmonary disease classification using CRX. We used the transfer learning technique for the base-CNNs. We analyzed some of the more well-known CNN architectures based on current literature, and have selected the six convolutional neural networks more adequate for the classification assignment (Table 2).

To exploit all the advantages of

the ensembles, we have evaluated the best way to combine the predictions. Predictions of the base CNNs are aggregated by a Voting Ensemble and a Stacking Ensemble (defined as a deep neural network). The Stacking Ensemble has the disadvantage of needing prior training, as opposed to the Voting Ensemble. We probed that our Stacking Ensemble has a slightly better performance with a 99% accuracy misclassifying 40 images of 2989 of the test images. The Voting Ensemble reaches an accuracy of 98 % (68 test images misclassified)

The Voting Ensemble shows slightly better performance than the Learning Ensemble, this is noticeable especially when it comes to evaluate a single image (Fig. 7). However, the performance on large image sets is comparable. This is because the burden of processing falls on the CNNs that have to evaluate the images. To increase the overall system performance for large image sets, different types of parallelization can be considered. System performance can be improved by packaging images in batches (batch sizes). Note that a very large batch size can lead to a more complex calculation. An even more effective way to increase the system acceleration is by running each of the CNN models in parallel, for this it would be necessary to increase the number of available GPUs, to perform a parallelization of all the prediction processes.

We analyzed the heatmaps of the ensembles (see Fig. 6), adapting the Grad-CAM tool with the aim of creating a mixed heatmap with all the base CNNs. This analysis showed that our system detects radiological findings into the CRXs. It also demonstrated that the case of asymptomatic patients is the one giving more misclassifications. Thus, we would need to involve experts to interpret the Chest X-Ray and to make more focused training in those cases.

## Conclusion

Computer-aided diagnosis systems are widely employed to assist radiologists and doctors, aiming at the different medical systems around the world, which are under a big volume of work due to the aging of the population and the increase in chronic diseases. In this work, we have achieved the successful diagnosis with very high accuracy of COVID-19, viral pneumonia, and tuberculosis by using CRX. Moreover, the analysis of the adapting Grad-CAM maps depicts that our system detects radiological findings in the CRXs. The work presented in this paper significantly reduces the cost of the CRX analysis in two ways. First, by reducing the required execution time in diagnosis. Second, professionals can be supported by assisted analysis, reducing the required study time per subject. As a continuation of this work, we are increasing the dataset with other lung diseases CRX, such as lung cancer and more types of pneumonia . Moreover, we want to develop a complete computer-aided remote and accessible diagnosis service using chest X-Ray.

## Data Availability

The used datasets were obtained from publicly open-source datasets from: COVID-19 Radiography Database https://www.kaggle.com/tawsifurrahman/covid19-radiography-database; Tuberculosis (TB) Chest X-Ray Database https://www.kaggle.com/tawsifurrahman/tuberculosis-tb-chest-xray-dataset.

## Footnotes

1 https://doi.org/10.5281/zenodo.6637854.
